# The association between physical exercise and emotional eating among college students: the mediating roles of coping style and emotion regulation

**DOI:** 10.3389/fnut.2026.1869296

**Published:** 2026-07-09

**Authors:** Cao Longan, Liu Hengxian, Zheng Zhirong, Wang Lei, Ran Zhengban

**Affiliations:** 1 School of Physical Education, Southwest University, Chongqing, China; 2 School of Athletic Performance, Shanghai University of Sport, Shanghai, China; 3 Teaching and Research Association, Guang'an Institute of Technology, Guangan, Sichuan, China

**Keywords:** coping style, emotion regulation, emotional eating, nutrition-related behavior, physical exercise

## Abstract

**Background:**

Emotional eating is a nutrition-related behavior closely linked to stress and emotion regulation. This study examined the association between physical exercise and emotional eating among college students and tested whether coping style and emotion regulation mediated this relationship.

**Methods:**

A cross-sectional survey was conducted among 2,871 college students in Southwest China. Validated questionnaires were used to assess physical exercise, coping style, emotion regulation, and emotional eating. Correlation, regression, and serial mediation analyses were conducted using SPSS and PROCESS Model 6, with demographic variables controlled.

**Results:**

Physical exercise was negatively associated with emotional eating. It was related to more positive coping style and cognitive reappraisal, and to less negative coping style and expressive suppression. Both coping style and emotion regulation were statistically associated with the relationship between physical exercise and emotional eating through independent and sequential mediation pathways.

**Conclusions:**

Physical exercise may be associated with lower emotional eating among college students. The findings suggest that coping style and emotion regulation may represent important psychological correlates of the association between physical exercise and emotional eating.

## 1 Introduction

### 1.1 Emotional eating

Emotional eating refers to the tendency to eat in response to emotional states rather than physiological hunger (1). It commonly occurs under stress, anxiety, sadness, loneliness, or other negative affective experiences and is often accompanied by an increased preference for palatable, energy-dense foods, particularly those high in sugar, fat, and refined carbohydrates (2, 3). Although eating may temporarily alleviate emotional discomfort, repeated reliance on food as a means of emotional regulation may contribute to unhealthy dietary patterns, excessive energy intake, poorer diet quality, and increased consumption of highly processed foods (4–6). Consequently, emotional eating has increasingly been recognized as an important nutrition-related behavioral risk factor with potential implications for long-term health. Beyond its nutritional implications, emotional eating has also attracted considerable attention in eating-disorder research because it represents a common maladaptive response to emotional distress and may contribute to a broader spectrum of disordered eating behaviors (7). Previous studies have linked emotional eating to binge-eating tendencies, loss-of-control eating, overweight and obesity, and increased vulnerability to eating-related psychopathology (8–12). Emotional eating may therefore represent not only a maladaptive response to emotional distress but also an early behavioral indicator of eating-related difficulties that warrant attention in prevention and health-promotion efforts.

College students constitute a population particularly vulnerable to emotional eating (13). During the transition from adolescence to early adulthood, students frequently experience academic stress, interpersonal challenges, lifestyle transitions, irregular routines, and greater autonomy over food choices (14). These circumstances may weaken structured eating habits and increase the likelihood that food is used as a strategy for managing emotional discomfort (15). As a result, emotional eating among college students should be understood as the product of interactions among emotional, behavioral, and lifestyle-related factors.

Previous studies have consistently identified negative affect as an important antecedent of emotional eating (16). However, emotional distress does not inevitably lead to emotion-driven eating, suggesting that individual differences in behavioral resources, coping capacities, and psychological regulation processes may influence whether negative emotions are translated into eating behavior (17). Identifying protective factors that reduce susceptibility to emotional eating is therefore important not only for understanding psychological wellbeing but also for informing nutrition-related health promotion, eating-behavior interventions, and the prevention of disordered eating among university students.

### 1.2 Physical exercise and emotional eating

Physical exercise is a key lifestyle behavior associated with both physical and mental health (18). Physical exercise refers to planned, structured, and repetitive bodily movement performed with the goal of improving or maintaining physical fitness. In addition to its role in energy expenditure and weight management, regular physical exercise has been associated with healthier dietary habits, improved appetite regulation, greater adherence to healthy lifestyles, and reduced engagement in health-risk behaviors (19, 20). Importantly, engaging in regular exercise requires individuals to set goals, maintain behavioral consistency, monitor progress, and overcome short-term discomfort in pursuit of longer-term benefits. Through these processes, physical exercise may strengthen self-control, attentional focus, decision-making, and self-regulation capacities (21, 22). These benefits are particularly relevant for college students, whose eating behaviors are often influenced by academic stress, emotional fluctuations, irregular schedules, and increasing independence in food-related decision-making (23).

From a nutrition and eating-behavior perspective, physical exercise may serve as a protective factor against emotional eating. Compared with general physical activity, which includes unstructured daily movements (e.g., walking or incidental activity), physical exercise more strongly reflects intentional behavior, self-regulation, and goal-directed action. These characteristics are particularly relevant to the present study because emotional eating has been linked to difficulties in self-control, emotion regulation, and adaptive coping (24). In addition, participation in organized sports may be influenced by external factors such as institutional schedules, team requirements, and social contexts, and may not fully capture individuals' autonomous behavioral choices (25). In contrast, regular physical exercise better represents a modifiable lifestyle behavior that can be independently adopted and maintained (26). From a practical perspective, focusing on physical exercise may provide more direct implications for health promotion, as it can be targeted through intervention programs aimed at improving self-regulation, coping strategies, and nutrition-related behaviors among college students. Previous studies have suggested that physically active individuals are less likely to rely on food as a means ofd coping with emotional distress and may exhibit healthier patterns of food choice and eating regulation (27, 28). Consequently, understanding the relationship between physical exercise and emotional eating may contribute to the development of nutrition-related health-promotion strategies and preventive approaches targeting eating-related difficulties among young adults. The relationship between physical exercise and emotional eating can also be interpreted within the framework of the Compensatory Carry-Over Action Model (CCAM). The CCAM proposes that health behaviors such as physical exercise and dietary behaviors are interconnected rather than independent, and may influence one another through shared psychological and self-regulatory mechanisms (29). Within this framework, engagement in physical exercise may generate positive carry-over effects that extend beyond physical activity itself and influence eating-related behaviors.

This perspective is particularly relevant to emotional eating because emotional eating represents a form of eating behavior that is strongly influenced by affective states and emotional regulation processes (30). Therefore, the association between physical exercise and emotional eating may reflect not only differences in energy balance but also broader behavioral and psychological processes that shape eating behavior. However, the mechanisms through which physical exercise may be associated with emotional eating remain insufficiently understood.

### 1.3 Coping style and emotional eating

Coping style refers to the cognitive and behavioral strategies that individuals use when dealing with stress, emotional demands, and challenging life events (31). Positive coping strategies typically involve problem solving, seeking social support, cognitive reframing, and constructive emotional expression, whereas negative coping strategies are characterized by avoidance, denial, self-blame, withdrawal, and passive endurance (32–35). These coping patterns influence not only psychological adaptation but also health-related behaviors, including eating behavior (36).

Coping style is particularly relevant to emotional eating because emotional eating is frequently conceptualized as a maladaptive coping response to emotional distress (37). Rather than addressing the source of stress directly, some individuals may use food consumption as a strategy for temporary emotional relief. Previous studies have suggested that individuals who rely on avoidant or negative coping strategies are more likely to engage in emotion-driven eating, binge-eating tendencies, and other disordered eating behaviors, whereas adaptive coping strategies may reduce the likelihood of using food as a means of emotional regulation (38, 39). Therefore, coping style may represent an important behavioral pathway linking emotional experiences to eating-related outcomes.

Among college students, effective coping may be especially important because academic demands, interpersonal challenges, and developmental transitions frequently generate emotional stress. Students who habitually use positive coping strategies may be better able to identify stressors, seek support, and implement constructive responses before emotional distress escalatesv (40). Such strategies may reduce the need to rely on food for emotional comfort. In contrast, students who predominantly use negative coping strategies may experience greater emotional burden and lower behavioral control, increasing their vulnerability to emotional eating and related eating difficulties (41).

Physical exercise may be associated with coping style. Regular participation in physical exercise provides opportunities for mastery experiences, self-regulation, routine formation, and social interaction, all of which may contribute to more adaptive responses to stress (42, 43). In addition, physical exercise itself may function as an alternative coping strategy, allowing individuals to regulate emotional arousal through behavioral engagement rather than through food-related coping behaviors. Consequently, physically active individuals may be more likely to adopt positive coping strategies and less likely to rely on maladaptive coping responses. When these resources are reflected in more adaptive coping strategies and reduced reliance on maladaptive coping, the tendency to engage in emotional eating may be lower. Therefore, coping style may represent a plausible mediating pathway linking physical exercise and emotional eating.

Although previous studies have linked coping style to mental health outcomes, relatively few studies have examined its role in the association between physical exercise and emotional eating among college students. Investigating coping style as a mediator may therefore help clarify whether the association between physical exercise and emotional eating is statistically related to students' stress-management patterns and may provide implications for interventions aimed at reducing emotional eating and preventing eating-related difficulties in university populations.

### 1.4 Emotion regulation as a mediating mechanism

Emotion regulation refers to the processes through which individuals influence the occurrence, intensity, duration, and expression of emotional experiences (44). Among the most widely studied strategies, cognitive reappraisal involves modifying the interpretation of an emotional situation before emotional responses fully develop, whereas expressive suppression refers to inhibiting emotional expression after emotional arousal has occurred (45, 46). Although suppression may reduce visible emotional reactions, it is generally considered less effective in alleviating internal emotional distress (47, 48).

Emotion regulation is particularly relevant to emotional eating because emotional eating is often triggered by difficulties in managing negative emotions (49). When emotional distress is not effectively regulated, food may become an easily accessible source of temporary comfort and emotional relief. Previous studies have shown that poorer emotion regulation is associated with higher levels of emotional eating, greater preference for energy-dense foods, less healthy food choices, and poorer dietary quality (50, 51). Over time, these eating patterns may contribute to excessive energy intake and other nutrition-related health risks. In contrast, adaptive strategies such as cognitive reappraisal may reduce the likelihood that negative emotions are translated into maladaptive eating behaviors (52).

Physical exercise may contribute to emotion regulation through both physiological and psychological pathways. Regular exercise has been associated with reduced emotional tension, enhanced positive affect, greater self-efficacy, and improved emotional resilience (53–55). These benefits may facilitate more adaptive emotion-regulation strategies and reduce reliance on food as a means of coping with emotional distress. Consequently, physically active individuals may be more likely to use cognitive reappraisal and less likely to depend on expressive suppression.

If physical exercise is associated with more adaptive emotion regulation, individuals may be less likely to use food as a substitute strategy for managing emotional distress and more likely to maintain healthier eating behaviors (56). However, the specific roles of cognitive reappraisal and expressive suppression in the association between physical exercise and emotional eating remain insufficiently understood among college students.

### 1.5 Serial mediation of coping style and emotion regulation

Coping style and emotion regulation are related but distinct psychological processes. Coping style reflects how individuals respond to stressors at a broader cognitive and behavioral level, whereas emotion regulation focuses on how emotional experiences are managed (57, 58). In daily life, the way students cope with stress may shape the strategies they use to regulate emotions (59). Positive coping may create conditions for cognitive reappraisal, while negative coping may be accompanied by emotional inhibition and suppression (60).

This relationship suggests a possible serial pathway from physical exercise to emotional eating. Physical exercise may first be associated with coping style (61); coping style may then influence emotion regulation (62); and emotion regulation may ultimately affect emotional eating (63). Few studies have simultaneously examined physical exercise, coping style, emotion regulation, and emotional eating among college students. Most existing research has focused either on the direct link between negative affect and eating behavior or on single mediating factors. This leaves an important gap in understanding how lifestyle behavior and psychological regulation jointly contribute to emotional eating. Addressing this gap may provide evidence for integrated interventions that combine physical exercise promotion, coping-skills training, emotion-regulation education, and nutrition guidance.

Based on the above literature, the present study examined the association between physical exercise and emotional eating among college students and further tested the mediating roles of coping style and emotion regulation. Specifically, positive coping style, negative coping style, cognitive reappraisal, and expressive suppression were included to explore whether these psychological factors independently and sequentially explain the relationship between physical exercise and emotional eating. Accordingly, the following hypotheses were proposed:

H1: physical exercise is negatively associated with emotional eating among college students.

H2: Coping style mediates the relationship between physical exercise and emotional eating.

H3: Emotion regulation may help explain the relationship between physical exercise and emotional eating.

H4: Coping style and emotion regulation may operate together in the relationship between physical exercise and emotional eating.

By testing these hypotheses, this study aims to clarify the psychological pathways linking physical exercise to emotional eating and to provide empirical evidence for nutrition-related health promotion strategies among college students.

## 2 Materials and methods

### 2.1 Participants and procedure

This cross-sectional study was conducted in accordance with the Declaration of Helsinki, approved by the Ethics Committee of Southwest University (Approval No. 20251119L1), and carried out following the STROBE guidelines. A random sampling method was used to recruit 3,000 college students from several well-known university in Southwest China. All participants were informed of the study purpose, voluntary nature of participation, confidentiality protection, and their right to withdraw before completing the questionnaire. Written informed consent was obtained from all participants. Data were collected using anonymous self-administered questionnaires. Different recruitment procedures were adopted according to the teaching arrangements across academic years to ensure feasibility while maintaining random sampling principles. For freshmen and sophomores, questionnaires were distributed by public physical education instructors through class-based random sampling and were collected on site after completion. For juniors and seniors, as compulsory physical education course requirements had already been completed, participants were recruited through random sampling. This approach was intended to include students with different educational experiences and lifestyle characteristics. However, because participation was voluntary and recruitment was conducted within a limited geographic region, the findings may not be fully representative of all Chinese college students. After completing the survey, participants received a small commemorative gift as a token of appreciation for their time and contribution. A total of 3,000 questionnaires were distributed. After excluding questionnaires with a completion time of less than 10 min, missing responses, or invalid answers, 2,871 valid questionnaires were retained for analysis, yielding a valid response rate of 95.7% (Supplementary Table 2).

The demographic characteristics of the participants are presented in Table 1. A total of 2,871 college students were included in the analysis. Among them, 1,359 were male (47.34%) and 1,512 were female (52.66%). Regarding grade distribution, 726 participants were freshmen (25.29%), 837 were sophomores (29.15%), 621 were juniors (21.63%), and 687 were seniors (23.93%). In terms of academic major, 1,470 students were from humanities and social sciences (51.20%), and 1,401 were from natural sciences (48.80%). Regarding place of residence, 1,542 participants came from rural areas (53.71%), while 1,329 came from urban areas (46.29%).

**Table 1:** Demographic characteristics.

Variable	Category	*N*	Percentage (%)	Cumulative percentage (%)
Gender	Male	1,359	47.34	47.34
	Female	1,512	52.66	100.00
Grade	Freshman	726	25.29	25.29
	Sophomore	837	29.15	54.44
	Junior	621	21.63	76.07
	Senior	687	23.93	100.00
Academic major	Humanities and social sciences	1,470	51.20	51.20
	Natural sciences	1,401	48.80	100.00
Place of residence	Rural area	1,542	53.71	53.71
	Urban area	1,329	46.29	100.00
Physical exercise level	Low level	1,536	53.50	53.50
	Moderate level	597	20.79	74.29
	High level	738	25.71	100.00

According to the classification criteria of physical exercise level, 1,536 students (53.50%) were classified as having a low level of physical exercise, 597 students (20.79%) as having a moderate level, and 738 students (25.71%) as having a high level. These results indicate that more than half of the participants reported low levels of physical exercise.

### 2.2 Materials

#### 2.2.1 Physical exercise

The study utilized the physical exercise Rating Scale (PARS-3), revised by Liang Deqing et al. (64), to assess the physical exercise level of university students. PARS-3 was selected because the present study aimed to assess habitual physical exercise participation rather than estimate total physical activity energy expenditure. Compared with the International Physical Activity Questionnaire (IPAQ), which quantifies physical activity using MET-based indicators, PARS-3 focuses on exercise intensity, duration, and frequency and provides an overall evaluation of exercise engagement level. Given that the theoretical framework of this study emphasized psychological and behavioral correlates of exercise participation, including coping style and emotion regulation, PARS-3 was considered appropriate for the present research objectives. Furthermore, PARS-3 has been widely used and validated among Chinese college students, supporting its applicability in this population. PARS-3 measures physical exercise across three dimensions: exercise intensity (A), duration per session (B), and exercise frequency (C). Each dimension is assessed by a single item rated on a 1–5 scale. The total physical exercise score is calculated as follows: Intensity × (Duration−1) × Frequency, yielding a score ranging from 0 to 100. Based on this score, participants are categorized into three exercise levels: low ( ≤ 19 points), moderate (20–42 points), and high (≥43 points). The scale has shown good reliability and validity, and was used in its original form in this study. The Cronbach's α coefficient in the present study was 0.84.

#### 2.2.2 Coping styles

Coping style was assessed using the Simplified Coping Style Questionnaire (SCSQ), developed by Jie (65). The SCSQ is designed to evaluate the strategies individuals commonly use when coping with stress and difficulties. The questionnaire consists of 20 items and includes two dimensions: positive coping style and negative coping style. Each item is rated on a 4-point scale ranging from 0 (“never used”) to 3 (“often used”). The total score for positive coping ranges from 0 to 36, with higher scores indicating a greater tendency to adopt positive coping strategies.

The SCSQ has been previously validated in Chinese populations and has demonstrated good reliability and validity. In the present study, the Cronbach's α coefficient for the questionnaire was 0.93, indicating excellent internal consistency.

#### 2.2.3 Emotion regulation questionnaire

Emotion regulation was assessed using the Emotion Regulation Questionnaire (ERQ), which measures two commonly used emotion regulation strategies: cognitive reappraisal and expressive suppression. Cognitive reappraisal refers to changing the interpretation of an emotional situation before the emotional response is fully generated, whereas expressive suppression refers to inhibiting outward emotional expression after emotional arousal has occurred. Participants rated each item according to the extent to which it described their usual emotion regulation strategy, with higher scores indicating a greater tendency to use the corresponding strategy.

In the present study, the Cronbach's α coefficients for the cognitive reappraisal and expressive suppression dimensions were 0.93 and 0.91, respectively, indicating excellent internal consistency.

#### 2.2.4 . Emotional eating

The emotional eating subscale of the Dutch Eating Behavior Questionnaire (DEBQ) was used as the primary outcome variable (66). A 5-point Likert scoring system was used, ranging from “strongly disagree” to “strongly agree,” with scores from 1 to 5. Higher scores indicate a greater tendency toward emotional eating. The subscales showed good reliability in Chinese youth (67), with Cronbach's α coefficients of 0.92.

### 2.3 Data analysis

Data were analyzed using SPSS 26.0 and the PROCESS macro version 3.5. The data presented in this study are available in the Supplementary materials. First, data quality was assessed through common method bias and normality tests. Harman's single-factor test was used to examine common method bias, and skewness and kurtosis values were used to assess normality. Descriptive statistics were then calculated for all variables, including physical exercise, coping style, emotion regulation, and emotional eating. Frequency analyses were used to describe participants' demographic characteristics. Independent samples *t*-tests and one-way analysis of variance (ANOVA) were conducted where appropriate to examine group differences across demographic variables. Pearson's two-tailed correlation analysis was performed to examine the associations among the main variables. Multiple linear regression analyses were conducted to test the predictive relationships among physical exercise, coping style, emotion regulation, and emotional eating, controlling for gender, grade, academic major, and place of residence. To test the hypothesized mediation effects, Model 6 of the PROCESS macro was used. Physical exercise was entered as the independent variable, emotional eating as the dependent variable, and coping style and emotion regulation as mediating variables. Considering the different roles of positive coping style, negative coping style, cognitive reappraisal, and expressive suppression, four separate serial mediation models were constructed. Gender, grade, academic major, and place of residence were included as covariates.

Indirect effects were estimated using 5,000 bootstrap resamples, and 95% bias-corrected confidence intervals were calculated. An indirect effect was considered significant when the confidence interval did not include zero.

## 3 Results

### 3.1 Common method bias test

Because all data in this study were collected using self-reported questionnaires, common method bias may be a potential concern. Harman's single-factor test was conducted to assess whether common method bias substantially affected the results. The results showed that eight factors with eigenvalues greater than 1 were extracted, and the largest factor explained 30.52% of the total variance, which was below the commonly recommended threshold of 40%. Therefore, common method bias was not considered to be a serious problem in this study.

In addition, skewness and kurtosis were used to evaluate whether the sample data approximately followed a normal distribution. As shown in Table 2, the absolute values of kurtosis for physical exercise, coping style, emotion regulation, emotional eating, and their subdimensions were all below 7, and the absolute values of skewness were all below 2. These results indicate that the data were approximately normally distributed and suitable for subsequent correlation, regression, and mediation analyses.

**Table 2:** Descriptive statistical analysis summary table.

Variable	Dimension	M	SD	Min	Max	Kurtosis	Skewness
Physical exercise	Total score	29.072	25.699	0	100	0.319	1.106
	Exercise intensity	3.235	1.049	1	5	−0.590	0.010
	Exercise duration	3.201	1.041	1	5	−0.641	0.077
	Exercise frequency	3.237	1.041	1	5	−0.480	−0.017
Coping style	Total score	51.351	7.627	32	72	0.316	0.302
	Positive coping style	34.033	7.992	15	48	−0.562	0.018
	Negative coping style	17.318	5.314	8	30	−0.556	0.016
Emotion regulation	Total score	40.385	8.933	18	64	−0.145	−0.020
	Cognitive reappraisal	25.650	10.089	7	42	−1.246	0.290
	Expressive suppression	14.735	7.073	4	28	−1.331	−0.248
Emotional eating	Emotional eating	36.549	10.511	15	61	−0.619	−0.057

### 3.2 Correlation analysis

Pearson's two-tailed correlation analysis was conducted to examine the associations among physical exercise, coping style, emotion regulation, and emotional eating among college students. As shown in Table 3, all study variables were significantly correlated with each other (*p* < 0.01).

**Table 3:** Correlation analysis summary table.

Variable	M	SD	1	2	3	4	5	6
1. Physical exercise	29.07	25.70	1					
2. Positive coping style	34.03	7.99	0.32^**^	1				
3. Negative coping style	17.32	5.31	−0.30^**^	−0.40^**^	1			
4. Cognitive reappraisal	25.65	10.09	0.26^**^	0.25^**^	−0.22^**^	1		
5. Expressive suppression	14.74	7.07	−0.32 ^**^	−0.26^**^	0.29^**^	−0.51^**^	1	
6. Emotional eating subscale	36.55	10.51	−0.41^**^	−0.40^**^	0.39^**^	−0.28^**^	0.32^**^	1

^**^p < 0.01.

Specifically, physical exercise showed moderate negative associations with emotional eating (*r* = −0.41), negative coping style (*r* = −0.30), and expressive suppression (*r* = −0.32), and moderate positive associations with positive coping style (*r* = 0.32) and cognitive reappraisal (*r* = 0.26). Emotional eating was moderately associated with positive coping style (*r* = −0.40), negative coping style (*r* = 0.39), expressive suppression (*r* = 0.32), and cognitive reappraisal (*r* = −0.28), suggesting that both coping style and emotion regulation may be relevant correlates of emotional eating among college students. These correlations were generally small to moderate in magnitude and provided preliminary evidence supporting the subsequent regression and mediation analyses. Given the large sample size, the interpretation focused not only on statistical significance but also on the magnitude and direction of the observed associations.

### 3.3 Multicollinearity diagnosis

Before conducting the regression and mediation analyses, multicollinearity among the main predictors was examined. As shown in Table 4, the variance inflation factor (VIF) values for physical exercise, positive coping style, negative coping style, cognitive reappraisal, and expressive suppression ranged from 1.229 to 1.453, all below the commonly accepted threshold of 5. The tolerance values ranged from 0.688 to 0.814, all above 0.30. These results indicate that multicollinearity was not a serious concern in the present study, and the variables were suitable for subsequent regression and mediation analyses.

**Table 4:** Multicollinearity diagnosis of the main predictors.

Variable	VIF	Tolerance
Physical exercise	1.229	0.814
Positive coping style	1.286	0.778
Negative coping style	1.274	0.785
Cognitive reappraisal	1.383	0.723
Expressive suppression	1.453	0.688

### 3.4 Regression models predicting coping style, emotion regulation, and emotional eating

As shown in Table 5, the overall regression model was significant (*R*^2^ = 0.502, *F* = 106.195, *p* < 0.01). Physical exercise demonstrated a moderate-to-large negative association with emotional eating in the initial model (β = −0.567, *p* < 0.001). Physical exercise was also moderately associated with coping style, showing a positive association with positive coping style (β = 0.405, *p* < 0.001) and a negative association with negative coping style (β = −0.398, *p* < 0.001). These findings suggest that higher levels of physical exercise are related to more adaptive coping patterns among college students.

**Table 5:** Regression models predicting coping style, emotion regulation, and emotional eating.

Outcome variable	Predictor	*R* ^2^	Adjusted *R*^2^	F	β	t
Emotional eating	Gender	0.353	0.349	103.558	−0.227	−8.584^**^
	Grade				0.223	8.201^**^
	Academic major				0.042	1.622
	Place of residence				0.008	0.312
	Physical exercise				−0.567	−20.398^**^
Positive coping style	Gender	0.177	0.173	40.931	0.056	1.879
	Grade				−0.260	−8.483^**^
	Academic major				−0.018	−0.598
	Place of residence				−0.062	−2.040^*^
	Physical exercise				0.405	12.903^**^
Negative coping style	Gender	0.181	0.177	42.069	−0.144	−4.837^**^
	Grade				0.227	7.428^**^
	Academic major				0.013	0.440
	Place of residence				0.108	3.536^**^
	Physical exercise				−0.398	−12.713^**^
Cognitive reappraisal	Gender	0.119	0.113	18.383	−0.018	−0.586
	Grade				−0.032	−0.954
	Academic major				−0.048	−1.561
	Place of residence				0.087	2.725^**^
	Physical exercise				0.166	4.522^**^
	Positive coping style				0.150	4.287^**^
	Negative coping style				−0.108	−3.096^**^
Expressive suppression	Gender	0.155	0.149	24.821	−0.027	−0.888
	Grade				0.003	0.085
	Academic major				0.017	0.574
	Place of residence				−0.048	−1.539
	Physical exercise				−0.217	−6.064^**^
	Positive coping style				−0.121	−3.538^**^
	Negative coping style				0.169	4.927^**^
Emotional eating	Gender	0.502	0.498	106.195	−0.183	−7.762^**^
	Grade				0.109	4.340^**^
	Academic major				0.027	1.155
	Place of residence				−0.010	−0.429
	Physical exercise				−0.337	−11.969^**^
	Positive coping style				−0.201	−7.555^**^
	Negative coping style				0.171	6.404^**^
	Cognitive reappraisal				−0.141	−5.194^**^
	Expressive suppression				0.130	4.705^**^

^*^p < 0.05, ^**^p < 0.01.

When physical exercise and coping style were entered as predictors of emotion regulation, physical exercise remained positively associated with cognitive reappraisal (β = 0.166, *p* < 0.001) and negatively associated with expressive suppression (β = −0.217, *p* < 0.001). In addition, positive coping style showed positive associations with cognitive reappraisal (β = 0.150, *p* < 0.001) and negative associations with expressive suppression (β = −0.121, *p* < 0.001), whereas negative coping style showed the opposite pattern. Although these associations were generally small to moderate in magnitude, they consistently supported the proposed relationships among coping style and emotion regulation strategies.

In the final model, physical exercise remained negatively associated with emotional eating (β = −0.337, *p* < 0.001), although the magnitude of the association was attenuated after coping style and emotion regulation variables were included. Positive coping style (β = −0.201, *p* < 0.001) and cognitive reappraisal (β = −0.141, *p* < 0.001) were associated with lower emotional eating, whereas negative coping style (β = 0.171, *p* < 0.001) and expressive suppression (β = 0.130, *p* < 0.001) were associated with higher emotional eating. Overall, physical exercise exhibited the largest standardized effect among the predictors, suggesting that it may represent an important behavioral correlate of emotional eating in this population.

### 3.5 Mediation analysis

Mediation analyses were conducted using Model 6 of the PROCESS macro version 3.5 in SPSS 26.0. Physical exercise was specified as the independent variable, and emotional eating was specified as the dependent variable. Because positive coping style, negative coping style, cognitive reappraisal, and expressive suppression may operate through different pathways, four separate serial mediation models were tested. Gender, grade, academic major, and place of residence were included as covariates in all models.

Indirect effects were estimated using bootstrap method with 5,000 resamples. Bias-corrected 95% confidence intervals were calculated, and an indirect effect was considered significant if the confidence interval did not include zero. All path coefficients were standardized.

#### 3.5.1 Mediation analysis involving coping style and cognitive reappraisal

The mediation effects involving coping style and cognitive reappraisal are presented in Table 6. The total effect of physical exercise on emotional eating was −0.435, of which 32.64% was explained by the indirect pathways. The direct effect remained substantial (67.59% of the total effect), indicating partial mediation.

**Table 6:** Mediation analysis involving coping style and cognitive reappraisal.

Path	Positive coping style					Negative coping style				
	Effect size	SE	95%CI		Proportion	Effect size	SE	95%CI		Proportion
			Boot LLCL	Boot ULCL				Boot LLCL	Boot ULCL	
Total mediating effect	−0.435	0.025	−0.485	−0.386	100.00%	−0.435	0.025	−0.485	−0.386	100.00%
Direct effect	−0.294	0.024	−0.341	−0.247	67.59%	−0.297	0.024	−0.345	−0.250	68.28%
Total indirect effect	−0.142	0.018	−0.178	−0.107	32.64%	−0.137	0.018	−0.174	−0.104	31.49%
Ind1	−0.090	0.015	−0.121	−0.063	20.69%	−0.084	0.014	−0.113	−0.058	19.31%
Ind2	−0.040	0.008	−0.057	−0.024	9.20%	−0.044	0.009	−0.064	−0.028	10.11%
Ind3	−0.012	0.003	−0.019	−0.006	2.76%	−0.009	0.003	−0.015	−0.005	2.07%
(C1): Ind1–Ind2	−0.050	0.017	−0.085	−0.018		−0.040	0.017	−0.074	−0.008	
(C2): Ind1–Ind3	−0.078	0.014	−0.107	−0.052		−0.075	0.013	−0.102	−0.051	
(C3): Ind2–Ind3	−0.028	0.009	−0.045	−0.011		−0.035	0.009	−0.055	−0.017	

Ind1 represents the pathway physical exercise → positive coping style/negative coping style → emotional eating; Ind2 represents the pathway physical exercise → cognitive reappraisal → emotional eating; Ind3 represents the pathway physical exercise → positive coping style/negative coping style → cognitive reappraisal → emotional eating.

Among the indirect pathways, the strongest contribution was observed for the pathway through coping style alone (Ind1), which accounted for 20.69% of the total effect. The pathway through cognitive reappraisal alone (Ind2) accounted for 9.20%, whereas the serial mediation pathway involving both coping style and cognitive reappraisal (Ind3) accounted for 2.76% of the total effect. All indirect effects were supported by bootstrap confidence intervals that did not include zero, indicating reliable mediation effects.

**Figure 1:**
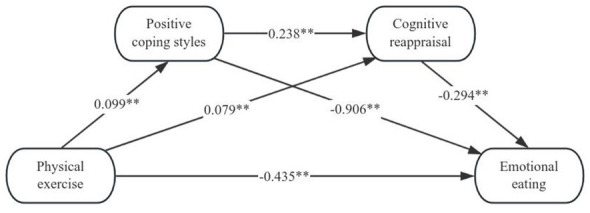
Serial mediation model of positive coping style and cognitive reappraisal. **p < 0.01.

**Figure 2:**
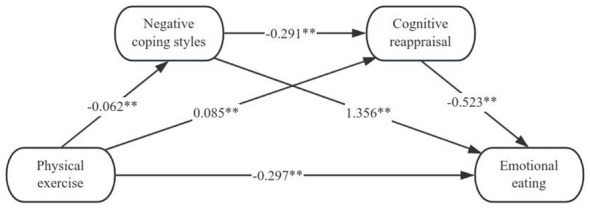
Serial mediation model of negative coping style and cognitive reappraisal. **p < 0.01.

Overall, these findings suggest that coping style represents the primary mediating mechanism linking physical exercise and emotional eating, whereas cognitive reappraisal contributes a smaller but meaningful additional indirect effect. The mediation model is illustrated in Figures 1, 2.

#### 3.5.2 Mediation analysis involving coping style and expressive suppression

The mediation effects involving coping style and expressive suppression are shown in Table 7. The total indirect effect accounted for 35.86% of the total association between physical exercise and emotional eating, whereas the direct effect accounted for 64.14%, indicating partial mediation.

**Table 7:** Mediation analysis involving coping style and expressive suppression.

Path	Positive coping style					Negative coping style				
	Effect size	SE	95%CI		Proportion	Effect size	SE	95%CI		Proportion
			Boot LLCL	Boot ULCL				Boot LLCL	Boot ULCL	
Total mediating effect	−0.435	0.025	−0.485	−0.386	100.00%	−0.435	0.025	−0.485	−0.386	100.00%
Direct effect	−0.279	0.024	−0.327	−0.232	64.14%	−0.290	0.024	−0.338	−0.243	66.67%
Total indirect effect	−0.156	0.019	−0.195	−0.121	35.86%	−0.145	0.018	−0.181	−0.111	33.33%
Ind1	−0.090	0.015	−0.120	−0.063	20.69%	−0.081	0.014	−0.109	−0.056	18.62%
Ind2	−0.054	0.010	−0.075	−0.036	12.41%	−0.051	0.010	−0.072	−0.034	11.72%
Ind3	−0.012	0.003	−0.019	−0.006	2.76%	−0.013	0.003	−0.020	−0.008	2.99%
(C1): Ind1–Ind2	−0.035	0.018	−0.071	−0.002		−0.030	0.017	−0.064	0.003	
(C2): Ind1–Ind3	−0.078	0.014	−0.107	−0.053		−0.068	0.013	−0.094	−0.045	
(C3): Ind2–Ind3	−0.042	0.010	−0.063	−0.024		−0.039	0.009	−0.058	−0.021	

Ind1 represents the pathway physical exercise → positive coping style/negative coping style → emotional eating; Ind2 represents the pathway physical exercise → expressive suppression → emotional eating; Ind3 represents the pathway physical exercise → positive coping style/negative coping style → expressive suppression → emotional eating.

The largest indirect effect was again observed for the pathway through coping style alone (Ind1), which explained 20.69% of the total effect. The pathway through expressive suppression alone (Ind2) accounted for 12.41%, while the serial mediation pathway involving coping style and expressive suppression (Ind3) explained 2.76% of the total effect. Bootstrap confidence intervals for all indirect effects excluded zero, supporting the robustness of the mediation findings.

**Figure 3:**
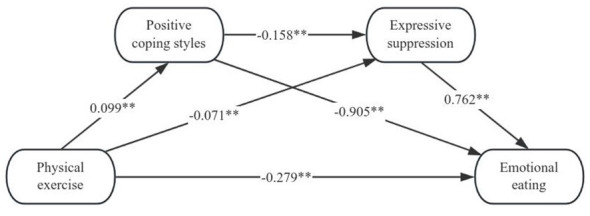
Serial mediation model of positive coping style and expressive suppression. **p < 0.01.

**Figure 4:**
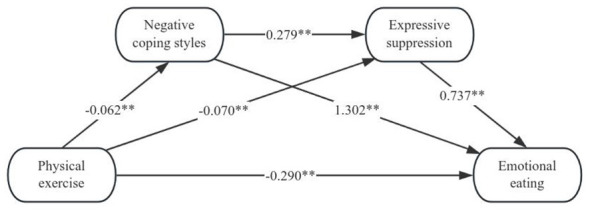
Serial mediation model of negative coping style and expressive suppression. **p < 0.01.

Taken together, these results suggest that both coping style and expressive suppression contribute to the association between physical exercise and emotional eating, although coping style appears to account for the largest proportion of the observed indirect effect. The mediation model is presented in Figures 3, 4.

## 4 Discussion

Overall, the present findings supported the proposed research hypotheses. Physical exercise was negatively associated with emotional eating among college students, and coping style and emotion regulation were showed significant indirect associations in the relationship between physical exercise and emotional eating. In addition, coping style and emotion regulation formed a serial mediation pathway, providing a more integrated statistical interpretation of how physical exercise may be associated with emotional eating. These results suggest that physical exercise may not only be directly related to lower emotional eating, but may also be associated with healthier eating-related psychological patterns, including greater positive coping style, lower negative coping style, greater cognitive reappraisal, and lower expressive suppression. The findings provide empirical evidence for the integration of physical exercise promotion, stress-coping training, emotion-regulation education, and healthy eating guidance in university health-promotion programs.

### 4.1 Direct association between physical exercise and emotional eating

The present study found a significant negative association between physical exercise and emotional eating among college students (68). After controlling for gender, grade, academic major, and place of residence, physical exercise remained a significant negative predictor of emotional eating. This finding suggesting that physical exercise may be an important lifestyle factor related to eating behavior and emotional regulation in university populations.

From the perspective of eating behavior, emotional eating is not primarily driven by physiological hunger. Rather, it reflects a tendency to eat in response to negative emotions, stress, or external cues. College students may be particularly vulnerable to this behavior because academic pressure, interpersonal adjustment, lifestyle changes, and limited self-management capacity can increase emotional distress and disrupt regular eating patterns. In this context, physical exercise may provide a non-food-based strategy for managing emotional discomfort, reducing students' reliance on food as an immediate source of comfort (69).

Several psychological and behavioral correlates may be related to this association. Physical exercise can improve mood, relieve psychological tension, enhance body awareness, and strengthen self-control. These characteristics may be associated with lower emotional eating tendencies under negative emotional states (70). Regular physical exercise may also increase students' attention to their health status and encourage more deliberate regulation of eating behavior.

### 4.2 Mediating role of coping style

The results further showed that coping style was statistically associated with the relationship between physical exercise and emotional eating through mediation pathways. Physical exercise was positively associated with positive coping style and negatively associated with negative coping style. In turn, positive coping style was negatively associated with emotional eating, whereas negative coping style was positively associated with emotional eating. These findings suggest that the relationship between physical exercise and emotional eating may be partly explained by how students respond to stress and emotional difficulties.

Coping style reflects the cognitive and behavioral patterns individuals use when facing stressful situations. Positive coping style generally involves active problem solving, seeking social support, positive reinterpretation, and appropriate emotional expression (71). These responses may help reduce the accumulation of negative emotions and decrease the need to use food as a means of stress relief. Negative coping style, such as avoidance, suppression, self-blame, or passive endurance, may prolong emotional distress and increase the likelihood that eating is used for short-term emotional comfort (72).

The association between physical exercise and coping style may be explained by psychological resources that tend to co-occur with regular physical exercise. Physical exercise is associated with higher levels of self-efficacy, psychological resilience, and stress tolerance, which in turn are linked to a greater tendency to adopt active and constructive responses when facing difficulties. At the same time, physical exercise often serves as a behavioral outlet for stress and emotional arousal, and this outlet is associated with reduced reliance on less adaptive coping patterns. Together, these observations support coping style as an important psychological factor linking physical exercise to emotional eating.

### 4.3 Mediating role of emotion regulation

Emotion regulation also mediated the relationship between physical exercise and emotional eating. Physical exercise was positively associated with cognitive reappraisal and negatively associated with expressive suppression. Cognitive reappraisal was negatively associated with emotional eating, whereas expressive suppression was positively associated with emotional eating (73).

Cognitive reappraisal is generally considered an adaptive emotion-regulation strategy because it involves changing the interpretation of an emotional situation before the emotional response is fully developed. Students who use cognitive reappraisal may be better able to reinterpret stressful events, reduce the intensity of negative affect, and avoid using food as a compensatory source of comfort (74). Expressive suppression, by contrast, may reduce outward emotional expression without effectively resolving internal emotional distress. When negative emotions remain unprocessed, eating may become an accessible and private way to obtain temporary relief.

Physical exercise may be associated with more adaptive emotion regulation through several pathways. Exercise can produce positive affective experiences, generate a sense of bodily pleasure, and provide an opportunity for emotional release. These characteristics of exercise may, in turn, be linked to more flexible cognitive appraisals and a lower tendency to suppress emotional expression. Therefore, students who engage in higher levels of physical exercise tend to report greater use of cognitive reappraisal and less reliance on expressive suppression. This pattern suggests that emotion regulation serves as a key explanatory pathway linking physical exercise to emotional eating.

### 4.4 Serial mediating role of coping style and emotion regulation

The serial mediation result is particularly meaningful in a statistical sense, as it suggests an indirect pathway from physical exercise to emotional eating through coping style and emotion regulation. Specifically, within the cross-sectional mediation model, physical exercise was associated with coping style (positive or negative), which in turn was associated with emotion regulation strategies (cognitive reappraisal or expressive suppression). These sequential associations were further linked to emotional eating. This pattern is consistent with a process whereby the link between physical exercise and emotional eating involves not a single psychological factor, but a chain of associations connecting stress coping and emotion regulation (75).

It is important to note that coping style and emotion regulation are conceptually distinct yet closely related in the context of stress. Coping style refers to broader patterns of cognitive and behavioral responses to stress, whereas emotion regulation focuses more specifically on the management of emotional experiences and expression (76). In the observed correlational pattern, a positive coping style was associated with more effective emotion regulation (e.g., greater use of cognitive reappraisal and less use of expressive suppression), which in turn was associated with lower emotional eating. Conversely, negative coping showed associations with expressive suppression and higher emotional eating (77). However, due to the cross-sectional design, these associations should not be interpreted as causal or temporal sequences.

Given the cross-sectional nature of this study, we reframe our interpretation as follows: physical exercise is associated with coping style and emotion regulation in a statistically sequential manner; this does not imply a temporal order. Instead, the findings indicate that the relationship between physical exercise and emotional eating may be statistically explained—at least in part—by a chain of associations involving coping style and emotion regulation.

## 5 Conclusions and recommendations

This study examined the association between physical exercise and emotional eating among college students in Southwest China and further explored the mediating roles of coping style and emotion regulation. Results showed a negative association between physical exercise and emotional eating, indicating that students who reported higher exercise levels tended to report lower emotional eating. In addition to this direct association, coping style and emotion regulation explained part of the relationship between physical exercise and emotional eating. Specifically, physical exercise was linked to more positive coping style, less negative coping style, greater use of cognitive reappraisal, and lower use of expressive suppression, which in turn were associated with reduced emotional eating tendencies.

These findings indicate that emotional eating should not be understood only as a dietary problem, but also as a behavioral expression of stress coping and emotion regulation patterns. Based on the above findings, the following considerations are proposed as references for university students in Southwest China:

First, based on the observed associations, physical exercise could be considered a lifestyle-related factor potentially relevant to emotional eating among university students. Given that emotional eating was associated with stress, negative affect, and difficulties in self-regulation, addressing emotional eating should not focus solely on dietary restriction or nutrition knowledge. Regular physical activity may represent an alternative behavior worth exploring for stress relief and emotion regulation, which could reduce reliance on food as a source of emotional comfort.

Second, coping-skills development may be incorporated into strategies targeting eating behaviors. Students could be supported in developing active coping strategies, such as problem solving, help-seeking, positive reframing, and appropriate emotional expression, while reducing reliance on avoidant or passive coping. Observations suggest that improving coping strategies may be relevant for lowering the tendency to use eating as a compensatory response to stress.

Third, emotion-regulation strategies, including enhancing cognitive reappraisal and reducing excessive expressive suppression, may be relevant in the study of emotional eating. Health promotion initiatives could exploring ways to help students identify emotional triggers for eating, distinguish physiological hunger from emotion-driven eating, and use alternative strategies instead of food-based regulation such as exercise, relaxation, communication, and mindfulness-based techniques.

Finally, the serial mediation analysis indicated potential value in considering multi-component approaches rather than single-component strategies. A model combining promotion of physical activity, coping-skills training, emotion-regulation strategies, and nutrition guidance may simultaneously relate to behavioral and psychological factors associated with emotional eating. In university settings, where academic stress and lifestyle transitions may increase vulnerability to emotion-driven eating, such multidisciplinary approaches could be particularly relevant for future research exploration.

## 6 Limitations and future research

Several limitations should be acknowledged. First, the cross-sectional design precludes causal inference. Although the proposed mediation model was theoretically grounded, longitudinal and intervention studies are needed to clarify the temporal sequence among physical exercise, coping style, emotion regulation, and emotional eating. Second, all variables were measured using self-reported questionnaires, which may introduce recall bias and social desirability bias. Furthermore, several nutrition- and lifestyle-related factors associated with emotional eating, including dietary restraint, perceived stress, and sleep quality, were not assessed and may have contributed to residual confounding. Future studies should include these variables to better understand emotional eating and related eating behaviors. Third, this study did not include several nutrition- and health-related variables, such as BMI, body weight status, dietary intake, sleep quality, perceived stress, anxiety, or depressive symptoms. These factors involve broader physiological, psychological, nutritional, and clinical domains and were beyond the primary scope of the present study. Future studies should include objective measures of physical exercise and more detailed dietary assessments to better understand the relationship between physical exercise and emotional eating. In addition, participants were recruited from universities in Southwest China, and participation was voluntary. Therefore, the representativeness of the sample may be limited, and selection bias cannot be completely excluded.

## Data Availability

The original contributions presented in the study are included in the article/Supplementary material, further inquiries can be directed to the corresponding authors.
