# Supplementary material for: The association between physical exercise and emotional eating among college students: the mediating roles of coping style and emotion regulation
Source: Front Nutr. 2026 Jul 9;13:1869296. doi: 10.3389/fnut.2026.1869296 (PMC13394457; doi:10.3389/fnut.2026.1869296)
Supplement: Supplementary file 2 [file Supplementary_file_2.pdf]

STROBE Statement—Checklist of items that should be included in reports of ***cross-sectional studies***

	Item No	Recommendation	Page No
**Title and abstract**	1	(*a*) Indicate the study's design with a commonly used term in the title or the abstract	Abstrat: Methods
		(*b*) Provide in the abstract an informative and balanced summary of what was done and what was found	Abstrat: Conclusions
**Introduction**			
Background/ rationale	2	Explain the scientific background and rationale for the investigation being reported	1 Introduction
Objectives	3	State specific objectives, including any prespecified hypotheses	1.5 Serial Mediation of Coping Style and Emotion Regulation
**Methods**			
Study design	4	Present key elements of study design early in the paper	Abstrat: Methods
Setting	5	Describe the setting, locations, and relevant dates, including periods of recruitment, exposure, follow-up, and data collection	2.1 Participants and procedure
Participants	6	(*a*) Give the eligibility criteria, and the sources and methods of selection of participants	2.1 Participants and procedure
Variables	7	Clearly define all outcomes, exposures, predictors, potential confounders, and effect modifiers. Give diagnostic criteria, if applicable	2.1 Participants and procedure
Data sources/ measuremen t	8*	For each variable of interest, give sources of data and details of methods of assessment (measurement). Describe comparability of assessment methods if there is more than one group	2 Materials and methods
Bias	9	Describe any efforts to address potential sources of bias	3.1. Common method bias test
Study size	10	Explain how the study size was arrived at	2.1 Participants and procedure
Quantitative variables	11	Explain how quantitative variables were handled in the analyses. If applicable, describe which groupings were chosen and why	2.1 Participants and procedure
Statistical methods	12	(*a*) Describe all statistical methods, including those used to control for confounding	2.1 Participants and procedure
		(*b*) Describe any methods used to examine subgroups and interactions	3.2 Correlation Analysis
		(*c*) Explain how missing data were addressed	2.1 Participants and procedure
		(*d*) If applicable, describe analytical methods taking account of sampling strategy	2 Materials and methods
		(*e*) Describe any sensitivity analyses	3.1. Common method bias test
**Results**			
Participants	13*	(a) Report numbers of individuals at each stage of study—eg	2.1 Participants and

		numbers potentially eligible, examined for eligibility, confirmed eligible, included in the study, completing follow-up, and analysed	procedure
		(b) Give reasons for non-participation at each stage	2.1 Participants and procedure
		(c) Consider use of a flow diagram	2.1 Participants and procedure
Descriptive data	14*	(a) Give characteristics of study participants (eg demographic, clinical, social) and information on exposures and potential confounders	2.1 Participants and procedure
		(b) Indicate number of participants with missing data for each variable of interest	2.1 Participants and procedure
Outcome data	15*	Report numbers of outcome events or summary measures	2.1 Participants and procedure
Main results	16	(*a*) Give unadjusted estimates and, if applicable, confounder-adjusted estimates and their precision (eg, 95% confidence interval). Make clear which confounders were adjusted for and why they were included	3.4. Regression Models Predicting Coping Style, Emotion Regulation, and Emotional Eating
		(*b*) Report category boundaries when continuous variables were categorized	3.4. Regression Models Predicting Coping Style, Emotion Regulation, and Emotional Eating
		(*c*) If relevant, consider translating estimates of relative risk into absolute risk for a meaningful time period	3.4. Regression Models Predicting Coping Style, Emotion Regulation, and Emotional Eating
Other analyses	17	Report other analyses done—eg analyses of subgroups and interactions, and sensitivity analyses	3.3. Multicollinearity Diagnosis
**Discussion**			
Key results	18	Summarise key results with reference to study objectives	4. Discussion
Limitations	19	Discuss limitations of the study, taking into account sources of potential bias or imprecision. Discuss both direction and magnitude of any potential bias	6. Limitations and Future Research
Interpretation	20	Give a cautious overall interpretation of results considering objectives, limitations, multiplicity of analyses, results from similar studies, and other relevant evidence	6. Limitations and Future Research
Generalisability	21	Discuss the generalisability (external validity) of the study results	6. Limitations and Future Research
**Other information**			
Funding	22	Give the source of funding and the role of the funders for the present study and, if applicable, for the original study on which the present article is based	4 Funding

*Give information separately for exposed and unexposed groups.

Note: An Explanation and Elaboration article discusses each checklist item and gives methodological background and published examples of transparent reporting. The STROBE checklist is best used in conjunction with this article (freely available on the Web sites of PLoS Medicine at http://www.plosmedicine.org/, Annals of Internal Medicine at http://www.annals.org/, and Epidemiology at http://www.epidem.com/). Information on the STROBE Initiative is available at www.strobe-statement.org.
